# Draft genome sequence of the New Jersey aster yellows strain of ‘*Candidatus* Phytoplasma asteris’

**DOI:** 10.1371/journal.pone.0192379

**Published:** 2018-02-06

**Authors:** Michael E. Sparks, Kristi D. Bottner-Parker, Dawn E. Gundersen-Rindal, Ing-Ming Lee

**Affiliations:** 1 USDA-ARS Invasive Insect Biocontrol and Behavior Laboratory, Beltsville, Maryland, United States of America; 2 USDA-ARS Molecular Plant Pathology Laboratory, Beltsville, Maryland, United States of America; Academia Sinica, TAIWAN

## Abstract

The NJAY (New Jersey aster yellows) strain of ‘*Candidatus* Phytoplasma asteris’ is a significant plant pathogen responsible for causing severe lettuce yellows in the U.S. state of New Jersey. A draft genome sequence was prepared for this organism. A total of 177,847 reads were assembled into 75 contigs > 518 bp with a total base value of 652,092 and an overall [G+C] content of 27.1%. A total of 733 protein coding genes were identified. This Whole Genome Shotgun project has been deposited at DDBJ/ENA/GenBank under the accession MAPF00000000. This draft genome was used for genome- and gene-based comparative phylogenetic analyses with other phytoplasmas, including the closely related ‘*Ca*. Phytoplasma asteris’ strain, aster yellows witches’- broom (AY-WB). NJAY and AY-WB exhibit approximately 0.5% dissimilarity at the nucleotide level among their shared genomic segments. Evidence indicated that NJAY harbors four plasmids homologous to those known to encode pathogenicity determinants in AY-WB, as well as a chromosome-encoded mobile unit. Apparent NJAY orthologs to the important AY-WB virulence factors, SAP11 and SAP54, were identified. A number of secreted proteins, both membrane-bound and soluble, were encoded, with many bearing similarity to known AY-WB effector molecules and others representing possible secreted proteins that may be novel to the NJAY lineage.

## Introduction

Phytoplasmas are cell wall-less bacterial pathogens infecting both plants and insects. They are transmitted by leafhoppers, planthoppers and psyllids, and cause several hundred economically important plant diseases worldwide. A vast array of diverse phytoplasma strains are distributed on all continents. Phylogenetic analysis based on 16S rRNA gene sequences indicated that phytoplasmas compose a large, discrete and monophyletic clade paraphyletic to the genus *Acholeplasma* in the Mollicutes class [1]. Because of the inability to readily cultivate these organisms in cell-free media, the provisional genus ‘*Candidatus* Phytoplasma’ and ‘*Candidatus* Phytoplasma spp.’ were proposed to accommodate their classification [2]. For simple and rapid classification of phytoplasmas, a scheme was proposed based on RFLP analysis of 16Sr RNA sequence, which to date includes 32 16S ribosomal (16Sr) groups and more than 200 subgroups [3,4]. More than 30 species have been assigned to the provisionary genus ‘*Candidatus* Phytoplasma’. Because of the difficulty to obtain cell-free, pure phytoplasma cultures, to date only six fully-assembled genomes [5–10] and eleven partial draft genomes [11–21] have been reported. In the aster yellows group 16SrI, complete genomes of ‘*Candidatus* Phytoplasma asteris’ strain AY-WB (aster yellows witches’-broom), belonging to subgroup 16SrI-A,and strains OY-M (onion yellows-M), and MBSP (maize bushy stunt phytoplasma) strain M3, both belonging to subgroup16SrI-B, and the draft genome of strain OY-V (onion yellows), belonging to subgroup 16SrI-B, were published [5,6,10,14].

Phytoplasma group 16SrI represents one of the most diverse groupings of phytoplasmas; it contains more than 15 subgroups [22], several of which are distributed worldwide (e.g., 16SrI-A, 16SrI-B and 16SrI-C). This group consists of numerous, genetically-diverse strains associated with a wide array of plant species as well as insect vectors. Symptoms caused by the 16SrI group phytoplasmas include virescence and phyllody (i.e., green discoloration and leaf-like flower petals), witches’-broom, small leaf and flower, leaf yellows, shoot proliferation, sterile fruit or seed production, and lethal yellowing [22]. Usually, similar symptoms are caused by closely related strains in a given 16SrI subgroup, in a given plant species. Both subgroup 16SrI-A phytoplasma strains, NJAY and AY-WB, cause severe lettuce yellows [6,23], although the symptoms they exhibit are not completely identical. AY-WB exhibits symptoms including induction of vein clearing, yellowing, stunting, witches’-broom, pigment loss or sterility of flowers, and necrosis in lettuce and yellowing, stunting, and witches’- broom formation in *Arabidopsis thaliana* [6]. In periwinkle, NJAY exhibits symptoms including phyllody, witches’-broom or bud proliferation, and yellowing. Both phytoplasma strains are transmitted by the aster leafhopper, *Macrosteles quadrilineatus* (formerly *Macrosteles fascifrons*) [6,23]. To better understand the pathogenic nature of closely related phytoplasmas, genomic sequencing approaches afford clearer insights into the genetic determinants underlying these organisms’ pathogenic capabilities. In this communication, we report the draft genome sequence of the ‘*Candidatus* Phytoplasma asteris’ strain NJAY (New Jersey aster yellows), which belongs to subgroup 16SrI-A [22] and is closely related to AY-WB phytoplasma. A comparative genome sequence analysis between these two and other strains is also reported.

## Results

A total of 347,686 genomic reads comprising 141,050,336 bases was generated from a Roche/454 sequencing library. The reference-based assembly incorporated a total of 177,847 reads (~51.2% of sequenced reads) into 75 contigs comprising 652,092 bases and exhibiting an N50 of 21,929 bp. Contig lengths ranged from 518 to 57,687 bp, and the assembly’s overall [G+C] was 27.1%. This Whole Genome Shotgun project has been deposited at DDBJ/ENA/GenBank under the accession MAPF00000000. The version described in this paper is version MAPF01000000. S1 Table summarizes the syntenic regions observed between the chromosomal genome of NJAY and that of the AY-WB and OY-M reference sequences used to guide assembly; 70 NJAY contigs were syntenic with AY-WB and 5 with OY-M. Although also used as reference sequences, no evident syntenic blocks were found between NJAY and either of the Australian (PAa) and strawberry lethal yellows (SLY) strains of ‘*Candidatus* Phytoplasma australiense’.

**Fig 1:**
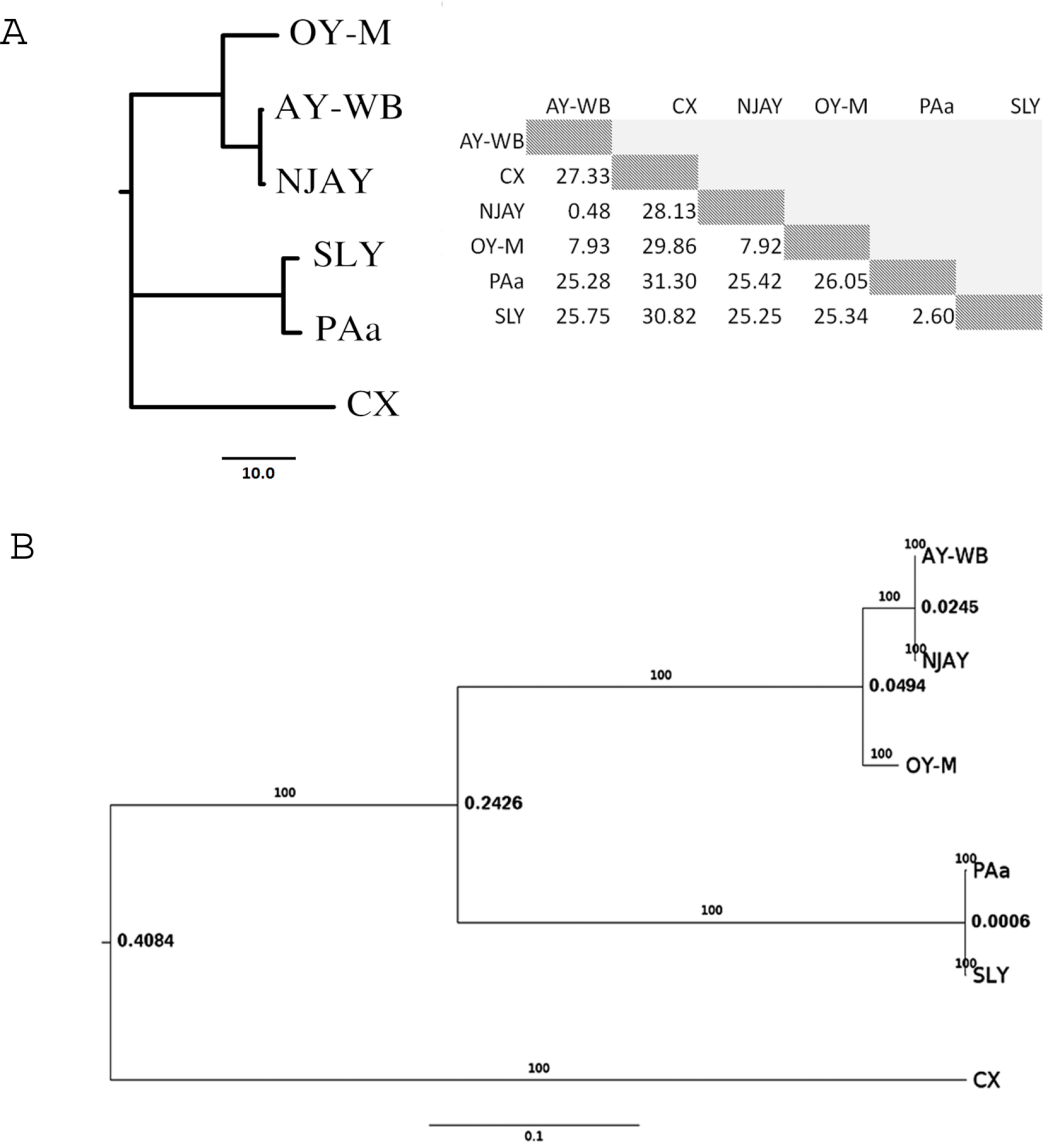
**A)** Neighbor-joining tree built using blast-based average nucleotide identity (ANIb) estimates calculated using JSpecies. Pairwise distances are provided in the inset table. As native ANIb values connote similarity measures; specifically, the proportion of identical residues among aligned segments expressed as a percentage; distance measures are obtained by taking complements with respect to 100%. ANIb considers similarity observed in all regions of the respective genomes where pairwise alignments using blastn are possible. **B)** Bayesian phylogeny produced using a codon-based model of DNA evolution applied to a concatemer of 29 reliably aligned, conserved protein-coding genes (6,618 codons in total). Branch lengths correspond to the number of nucleotide substitutions per codon. Posterior probabilities of the inferred branching patterns are indicated on the tree.

Phylogenetic analysis based on the ANIb measure of genomic similarity demonstrated the very close relatedness of the NJAY and AY-WB ‘*Ca*. Phytoplasma asteris’ strains (Fig 1A). Of 733 protein-coding genes identified in the NJAY genome, all but seven were demonstrably homologous to proteins previously identified in other phytoplasmas. In particular, none of these seven latter genes had hits per blastp in any of the annotated protein sets for AY-WB, OY-M, PAa, SLY or CX available at GenBank; however, all seven were identified in genomic sequence for AY-WB and one was identified in genome data for all the reference taxa. The sequence encoded in genomes for all reference phytoplasmas appears to encode a 16S rRNA gene, and notwithstanding its similarity to NCBI NR proteins from other bacterial species, very likely corresponds to a false positive protein-coding gene prediction by Prodigal. Five of the genes (three of which were exact copies encoded on separate contigs in NJAY) exhibited somewhat weak hits to non-phytoplasmal bacterial proteins; when using underlying coding sequences for these as blastx queries, one of these exhibits a match to a dihydropteroate synthase identified in various phytoplasmas, likely indicating that the NJAY sequence has either sustained a missense mutation or that an error may be present in the draft genome sequence. The seventh protein had a strong match to a *Vigna angularis* protein; as this sequence is also present in the AY-WB genome, whether it corresponds to an erroneously retained contaminant green plant sequence in the assembly or an instance of horizontal gene transfer remains to be elucidated. Sequence data for these genes are provided in S2 Table.

Protein-level sequence clustering identified a total of 37 clusters containing precisely one protein from all six taxa considered; eight of these clusters were discarded over one or more sequences having considerable discrepancies in length relative to the cluster consensus. For each of the 29 remaining genes, their concatenated, nucleotide-level alignments resulted in 19,854 reliably aligned, presumably orthologous nucleotide positions in total (6,618 codons; see Table 1). Consistent with observations obtained using genome-level data, the phylogram produced with gene-level data supports the notion of very close relatedness between NJAY and AY-WB, and is in accord with overall expectations for phylogenetic placement of the remaining taxa (Fig 1B).

**Table 1:** Conserved genes present as single copies in AY-WB, CX, NJAY, OY-M, PAa and SLY.

Gene	Aligned Codons
30S ribosomal protein S5	179
30S ribosomal protein S7	156
30S ribosomal protein S8	131
30S ribosomal protein S10	107
30S ribosomal protein S12	139
30S ribosomal protein S14	89
30S ribosomal protein S17	96
50S ribosomal protein L11	141
50S ribosomal protein L16	140
50S ribosomal protein L21	102
50S ribosomal protein L27	98
50S ribosomal protein L28	62
50S ribosomal protein L32	66
50S ribosomal protein L34	44
50S ribosomal protein L36	38
2-oxoisovalerate dehydrogenase subunit beta	326
6-phosphofructokinase	333
dipeptide/oligopeptide/nickel ABC transporter ATP-binding protein	267
elongation factor G	688
elongation factor Tu	394
enolase	430
fructose-1,6-bisphosphate aldolase, class II	252
glucose-6-phosphate isomerase	427
malate dehydrogenase	390
phosphoglycerate kinase	402
propanediol utilization protein	193
ribonucleotide-diphosphate reductase	351
type I glyceraldehyde-3-phosphate dehydrogenase	337
UMP kinase	240
	6,618

The number of reliably aligned (and presumably orthologous) codons contributed by each to the concatenated gene sequence used for phylogenetic analysis is indicated. (Note that although OY-M has two nominal ribonucleotide-diphosphate reductase genes, WP_011160430.1 and WP_041624910.1, these exhibit entirely dissimilar biosequences.)

Evidence for the existence of NJAY plasmids homologous to the pAYWB-I, pAYWB-II, pAYWB-III and pAYWB-IV plasmids of AY-WB was obtained (see Table 2). However, *ab initio* gene finding did not detect any substantive, uninterrupted reading frames on these plasmid sequences. In addition to plasmids, evidence for existence of the PMU1 (potential mobile unit-1) genetic element in NJAY, based on templated sub-assembly, was observed (see Table 2). Comparison of the four AY-WB PMUs with the overall NJAY chromosomal assembly demonstrated that at least 39 contigs exhibited significant hits with one or more of these genetic elements (see Table 3).

**Table 2:** Sub-assembled extrachromosomal NJAY sequences.

AY-WB Reference	Length of AY-WB Genetic Element	Positions of Shared Homology with NJAY	Length of NJAY Contig	Length of Match	Percent Similarity
pAYWB-I	3972	1..2793	2788	2810	94
		2933..3121	180	180	89
		3564..3972	417	418	95
pAYWB-II	4009	1271..2638	1368	1366	92
		2914..3624	702	626	97
pAYWB-III	5104	1..5104	5105	5128	98
pAYWB-IV	4316	1130..2586	1448	1467	92
		2770..3362	596	525	96
		3655..4002	358	325	97
PMU1	20,093	1..2699	2698	2693	98
		2981..20,093	17,104	17,150	98

Sequences similar to non-chromosomal genetic elements present in AY-WB, as well as PMU1 (i.e., potential mobile unit 1, a known AY-WB gene cluster that also exists in a circular, extrachromosomal form) are indicated. Length is given is base pairs.

**Table 3:** Contigs from NJAY chromosomal assembly exhibiting hits to PMUs of the AY-WB chromosome.

	PMU1	PMU2	PMU3	PMU4
MAPF01000003.1	x		x	x
MAPF01000004.1	x			
MAPF01000008.1		x		
MAPF01000010.1	x	x	x	x
MAPF01000011.1	x		x	x
MAPF01000014.1	x	x	x	x
MAPF01000016.1	x			x
MAPF01000019.1	x		x	x
MAPF01000022.1	x		x	x
MAPF01000023.1	x	x	x	x
MAPF01000024.1	x	x	x	x
MAPF01000025.1	x	x	x	x
MAPF01000026.1	x	x	x	x
MAPF01000027.1	x	x	x	
MAPF01000028.1	x		x	x
MAPF01000035.1	x			
MAPF01000037.1	x		x	
MAPF01000038.1	x	x	x	x
MAPF01000039.1	x	x	x	x
MAPF01000040.1		x		
MAPF01000042.1	x	x	x	x
MAPF01000045.1	x	x	x	x
MAPF01000046.1		x		
MAPF01000047.1	x		x	x
MAPF01000048.1	x	x	x	x
MAPF01000049.1	x	x	x	x
MAPF01000050.1	x		x	x
MAPF01000051.1	x		x	x
MAPF01000052.1	x		x	x
MAPF01000062.1	x		x	x
MAPF01000063.1	x	x	x	x
MAPF01000064.1	x	x	x	x
MAPF01000067.1	x	x	x	x
MAPF01000068.1	x		x	x
MAPF01000069.1			x	
MAPF01000072.1	x		x	
MAPF01000073.1	x		x	
MAPF01000074.1	x			x
MAPF01000075.1	x		x	x

Contigs exhibiting hits to all four reference PMUs are highlighted with a grey background.

Of the 27 potential secreted membrane proteins identified in NJAY, 19 were supported by homology with genes previously documented in AY-WB: nine such proteins were highly similar, one was moderately similar and nine were somewhat similar (see Table 4). Three of the 19 homology-supported genes were predicted to contain a signal peptide and one or more transmembrane helices by both computational model-based methods considered. Eight proteins not supported by extrinsic evidence were predicted as being secreted and membrane-bound, though none of these was simultaneously predicted as such by both methods.

**Table 4:** Candidate cell wall membrane-anchored, secreted proteins in NJAY.

Gene	Homology Info	Phobius?	SignalP+TMHMM?
contig00002_3	AYWB_006 (76.8%)		
contig00002_16	AYWB_013 (62.2%)		
contig00002_20	AYWB_016 (100.0%)	yes	yes
contig00010_12	AYWB_053 (76.7%)		yes
contig00018_12	AYWB_114 (100.0%)	yes	yes
contig00039_26	AYWB_256 (78.5%)		
contig00063_10	AYWB_320 (100.0%)		yes
contig00075_6	AYWB_395 (33.3%)		
contig00081_2	AYWB_413 (100.0%)		yes
contig00081_3	AYWB_414 (74.9%)		yes
contig00081_5	AYWB_415 (93.1%)	yes	
contig00082_6	AYWB_432 (67.8%)		
contig00084_8	AYWB_477 (100.0%)		yes
contig00084_34	AYWB_502 (75.1%)	yes	yes
contig00089_11	AYWB_530 (34.3%)		
contig00090_3	AYWB_534 (95.3%)		yes
contig00090_14	AYWB_544 (100.0%)		yes
contig00090_32	AYWB_561 (98.9%)		yes
contig00092_29	AYWB_599 (97.0%)		yes
contig00007_2	none	yes	
contig00010_10	none	yes	
contig00010_11	none	yes	
contig00074_16	none	yes	
contig00082_2	none	yes	
contig00096_12	none	yes	
contig00002_2	none		yes
contig00074_11	none		yes

Sequence similarity with apparent AY-WB homologs known to contain a signal peptide and at least one transmembrane helix is shown. Also indicated is whether the model-based methods of Phobius and SignalP+TMHMM predicted the protein in question as being a membrane-bound secreted protein. Protein records supported by all three criteria are highlighted with a grey background.

Forty-three NJAY proteins were observed to share sequence similarity with known soluble (i.e., non-membrane bound) secreted proteins in AY-WB (see Table 5). Of these cognate pairs, 18 were highly similar, six moderately similar and 19 somewhat similar. Ten of these NJAY proteins were identified as containing a signal sequence (but no transmembrane helices) by both of the predictive methods utilized and 15 by only one such method. Twenty-two proteins were flagged as being secreted and soluble exclusively by model-based methods, two of which were labeled as such by both programs (see Table 6).

**Table 5:** Homology-supported candidate NJAY secreted proteins to be released as soluble biomolecules.

Gene	Homology Info	Phobius?	SignalP+TMHMM?
contig00074_19	SAP08 (AYWB_387: 76.5%)	yes	yes
contig00074_6	SAP09 (AYWB_376: 100.0%)		yes
contig00073_13	SAP11 (AYWB_370: 100.0%)	yes	yes
contig00101_1	SAP13 (AYWB_640: 100.0%)	yes	yes
contig00096_11	SAP15 (AYWB_624: 100.0%)		yes
contig00015_15	SAP19 (AYWB_073: 100.0%)		yes
contig00037_14	SAP20 (AYWB_645: 35.6%)		
contig00003_7	SAP21 (AYWB_022: 59.2%)	yes	yes
contig00078_3	SAP22 (AYWB_275: 53.4%)		
contig00026_1	SAP25 (AYWB_148: 25.7%)		
contig00021_24	SAP26 (AYWB_146: 35.7%)		
contig00019_4	SAP27 (AYWB_127: 38.3%)		
contig00090_33	SAP34 (AYWB_562: 100.0%)		yes
contig00092_17	SAP35 (AYWB_588: 92.0%)		
contig00120_1	SAP36 (AYWB_189: 33.3%)		
contig00037_40	SAP39 (AYWB_212: 100.0%)		yes
contig00039_1	SAP40 (AYWB_236: 100.0%)		yes
contig00039_2	SAP41 (AYWB_237: 100.0%)		yes
contig00040_1	SAP42 (AYWB_258: 80.8%)	yes	yes
contig00015_31	SAP43 (AYWB_259: 18.9%)		
contig00061_2	SAP45 (AYWB_294: 47.3%)		
contig00069_2	SAP47 (AYWB_342: 85.5%)		yes
contig00008_3	SAP48 (AYWB_340: 42.4%)	yes	yes
contig00008_2	SAP49 (AYWB_339: 28.7%)	yes	yes
contig00082_7	SAP50 (AYWB_433: 100.0%)		yes
contig00062_1	SAP51 (AYWB_295: 100.0%)	yes	yes
contig00047_3	SAP52 (AYWB_263: 76.6%)		yes
contig00038_2	SAP53 (AYWB_225: 95.2%)		yes
contig00038_1	SAP54 (AYWB_224: 61.3%)		yes
contig00037_32	SAP55 (AYWB_203: 81.4%)	yes	yes
contig00073_10	SAP56 (AYWB_367: 100.0%)	yes	yes
contig00021_23	SAP59 (AYWB_145: 96.8%)		yes
contig00027_1	SAP60 (AYWB_152: 29.9%)		
contig00034_2	SAP61 (AYWB_169: 80.5%)		
contig00038_6	SAP62 (AYWB_229: 57.3%)		
contig00039_12	SAP63 (AYWB_245: 72.9%)		
contig00064_1	SAP65 (AYWB_329: 99.7%)		yes
contig00073_9	SAP66 (AYWB_366: 100.0%)		yes
contig00073_11	SAP67 (AYWB_368: 93.3%)		yes
contig00073_12	SAP68 (AYWB_369: 95.4%)		yes
contig00084_11	SAP69 (AYWB_480: 97.3%)	yes	
contig00089_7	SAP70 (AYWB_529: 45.6%)		
contig00105_24	SAP72 (AYWB_667: 65.8%)		

Sequence similarity with apparent AY-WB homologs known to contain a signal peptide (but no transmembrane helices) is shown. Also indicated is whether the model-based methods of Phobius and SignalP+TMHMM predicted the protein in question as harboring a signal peptide. Protein records supported by all three criteria are highlighted with a grey background.

**Table 6:** Non-membrane bound secreted protein candidates in NJAY lacking evident homology with such proteins in AY-WB.

Gene	Phobius?	SignalP+TMHMM?
contig00039_26	yes	yes
contig00105_16	yes	yes
contig00018_23	yes	
contig00021_16	yes	
contig00021_3	yes	
contig00034_1	yes	
contig00039_5	yes	
contig00062_2	yes	
contig00069_5	yes	
contig00078_2	yes	
contig00084_19	yes	
contig00090_29	yes	
contig00092_27	yes	
contig00105_9	yes	
contig00004_1		yes
contig00015_25		yes
contig00029_3		yes
contig00038_7		yes
contig00075_5		yes
contig00089_10		yes
contig00089_6		yes
contig00105_25		yes

Whether the model-based methods of Phobius and SignalP+TMHMM predicted the protein in question as harboring a signal peptide is shown. Protein records supported by both methods are highlighted with a grey background.

**Fig 2:**
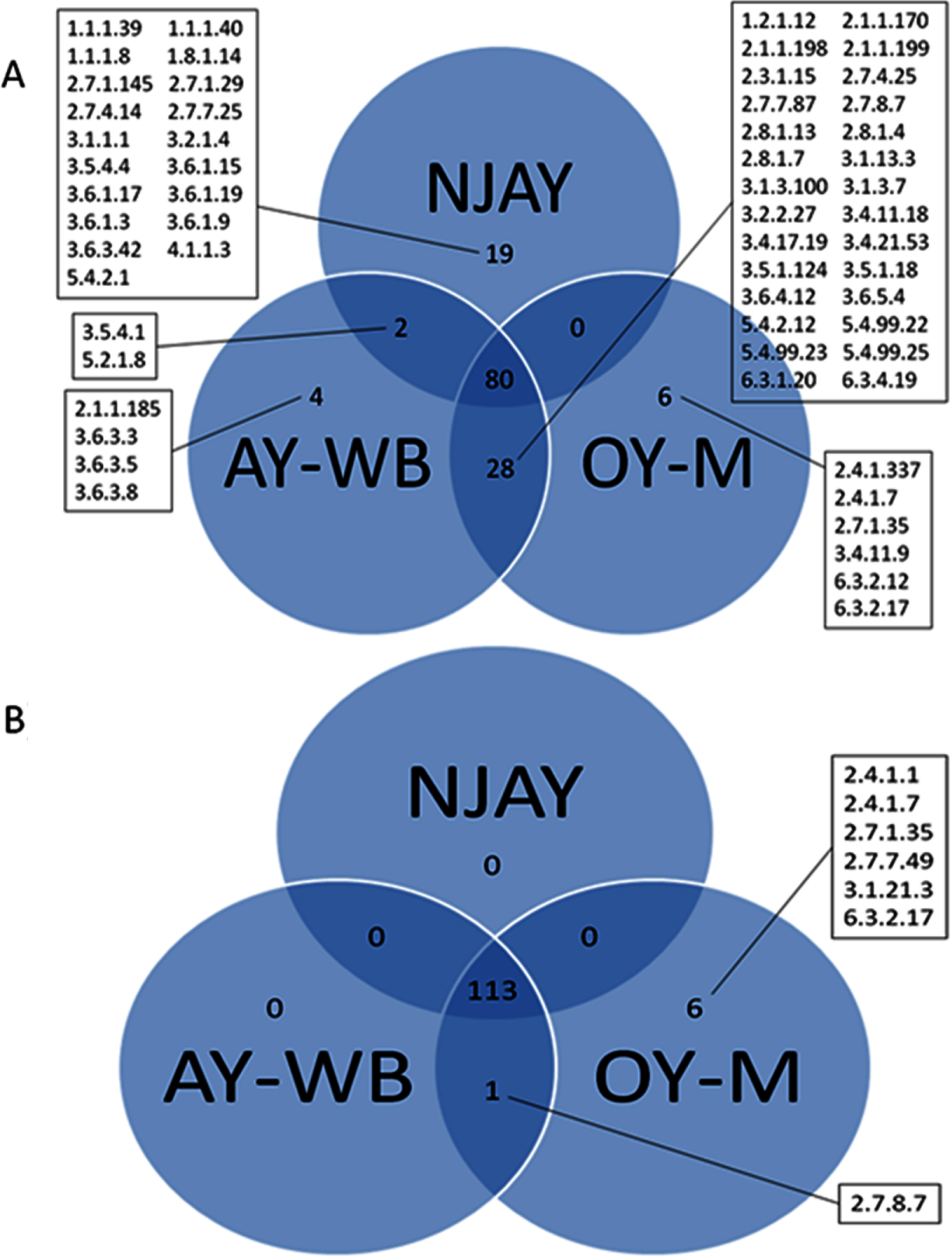
**A)** Venn diagram comparing species-specific Enzyme Commission (EC) code listings. Enzymatic category identifiers were obtained from NJAY using Prodigal-predicted proteins annotated with Blast2GO, and from AY-WB and OY-M using annotations currently available in KEGG per EC2KEGG. EC codes not mutual to all three taxa are indicated. **B)** As above, but using EC codes for AY-WB and OY-M following reannotation of their respective protein-coding genes via Prodigal and Blast2GO.

Comparison of the enzymatic capacities of NJAY, AY-WB and OY-M using prior annotations resulted in the identification of 139 unique Enzyme Commission (EC) codes overall, of which 80 (~57.6%) were mutually present in all three taxa (Fig 2A). Thirty (~21.6%) were found in pairwise, but not three-way, comparisons and 29 (~20.9%) were specific to a particular species. A similar three-fold comparison utilizing updated protein predictions demonstrated that 113 EC codes were mutually shared, one enzymatic category was exclusive to AY-WB and OY-M, and six appeared exclusive to the OY-M lineage (Fig 2B). The comprehensive listing of enzymatic capacities identified, as well as recommended names for each EC code encountered, is available in S3 Table.

## Discussion

Early hybridization studies suggested that a high degree of similarity exists between the genomes of subgroup 16SrI-A phytoplasmas [24]. Comparative genomics results of NJAY and AY-WB strains presented in this study corroborate those earlier findings, demonstrating that high levels of similarity at the molecular level are indeed present between strains within the same subgroup. Phylograms derived from similarity measures based on both whole-genome and protein-coding gene sequences demonstrate the very close placement of NJAY and AY-WB within a broader set of representative phytoplasma taxa. The phylogenetic placement of all taxa considered was consistent with *a priori* expectations. NJAY and AY-WB exhibit roughly 0.5% dissimilarity at the nucleotide level among their shared genomic segments, which is approximately five-fold less than the levels observed between two strains of ‘*Candidatus* Phytoplasma australiense’, SLY and PAa (Fig 1A). Interestingly, among conserved orthologous genes, the estimated number of nucleotide substitutions per codon between NJAY and AY-WB is roughly 40-fold higher than that between SLY and PAa (Fig 1B), which may reflect the fact that far fewer sites were considered for the concatenated coding sequence comparison than for full genome comparisons (and thus may reflect some inherent degree of small sample bias) or that NJAY, AY-WB or both are exposed to environments more favorable to diversifying selection at the coding sequence level than those to which SLY and PAa are subjected.

This study also demonstrates that, like AY-WB, NJAY harbors plasmids and other mobile genomic elements (e.g., PMU1), which may contribute to genomic plasticity. As such, these elements can accommodate the gradual accumulation of lineage-specific changes over time, resulting in modifications to genomic structure that in turn may effect changes in phenotypic expression (i.e., symptom variation in infected host plants). In AY-WB, these specific plasmids are thought to encode genetic determinants of pathogenicity, although *ab initio* gene finding routines did not suggest any intact genes harbored by the NJAY plasmids. However, tblastn comparisons between known AY-WB plasmid-borne gene sequences and the NJAY plasmids suggested that these genes may exist in NJAY, but the matches observed were fairly weak and fragmentary (data not shown). More intensive, plasmid-targeted sequencing in NJAY should resolve whether these fragments truly correspond to pseudogenes or whether they are an artifact of incomplete and/or inaccurate plasmid sequencing and assembly.

Thirty-nine NJAY chromosomal contigs bore similarity to one or more of the four potential mobile units (PMUs) identified in AY-WB. Given the repetitive nature of these genetic elements and the fragmentary character of the draft NJAY genome sequence produced here, a complete characterization of NJAY’s PMU content was not feasible. The MAPF01000026.1 contig in particular exhibited very high similarity levels with each of the four AY-WB PMUs (e.g., it exhibited 98% similarity with the 3’-most 14,495 bases of the 20,093 bp AY-WB PMU1 element), suggesting it encodes the NJAY PMU most closely related to its AY-WB counterparts.

In AY-WB, the chromosome-encoded, secreted and soluble proteins SAP11 and SAP54 were demonstrated to accumulate in cellular nuclei of infected plants and to function as virulence factors (effectors): SAP11 induced axillary shoot proliferation and SAP54 caused floral virescence and phyllody in infected plants (*Arabidopsis thaliana*) [25,26]. Apparent NJAY orthologs to SAP11 and SAP54 were identified which shared 100.0% and 61.3% global sequence similarity, respectively, with their AY-WB counterparts. However, the draft NJAY SAP54 ortholog sequence is only 83 aa long (vs 124 residues for AY-WB SAP54) and may very likely be incomplete and/or incorrect at its C-terminus: the first 71 amino acids of this protein exhibit perfect identity with those of its AY-WB counterpart. Indeed, if a putative guanine insertion mutation (position 47 of contig MAPF01000027.1) that apparently effects a missense mutation is disregarded, the first 86 residues of AY-WB SAP54 are present with perfect identity on the encoding contig’s reverse strand, at which point the 5’-terminus is encountered and any additional coding sequence remains unknown. These results suggest that the NJAY SAP54 is likely much more similar to the AY-WB SAP54 than the current draft genome sequence suggests. More specific sequencing of this genomic region will be necessary to disambiguate whether NJAY harbors a truncated, perhaps non-functional, copy of this gene or a version highly similar to that of AY-WB.

The NJAY genomic DNA sequenced in this study was prepared from symptomatic periwinkle plants (specifically, plants exhibiting phyllody and witches’-broom) infected with NJAY phytoplasma. A number of grafts maintained over 20 years in the USDA-ARS greenhouse facility have been observed exhibiting various phenotypic symptoms in infected periwinkle. A distinct NJAY lineage has been propagated in periwinkle plants exhibiting shoot proliferation and small white flowers (lack of floral virescence and phyllody). Further exploration will be needed to determine whether variation in secreted and soluble proteins, including those homologous to AY-WB SAP11 and SAP54, in this NJAY lineage was responsible for these specific phenotypic changes of the infected periwinkle plants.

Comparisons of the enzymatic capacities embedded in protein repertoires of NJAY, AY-WB and OY-M varied depending on whether pre-existing annotations available from the KEGG database were used for the latter two taxa or whether these were reannotated using the Prodigal gene finder (see Fig 2). The first comparison suggests a potentially greater degree of metabolic distinctiveness exists among these species than does the second (80 ÷ 139 ≈ 57.5% mutual overlap based on prior annotations *versus* 113 ÷ 120 ≈ 94.2% using revised data). However, the greater concordance achieved through use of updated annotation data suggests that NJAY and AY-WB exhibit minimal enzymatic differences (only one non-shared enzymatic category), and OY-M only a moderate level of distinctiveness (six unique EC codes). Indeed, the single discrepancy observed between NJAY and AY-WB, EC 2.7.8.7 (encoding holo-[acyl-carrier-protein] synthases), may be an artifact of errant genome sequencing and/or assembly: The AY-WB protein bearing this EC code was aligned to the draft NJAY assembly using tblastn, and the first twelve and last 103 residues of this 126 aa protein (i.e., ~91.3%) aligned with perfect similarity to bases 13,291 through 13,326 and 13,359 through 13,667, respectively, of contig MAPF01000043.1. Further experimental testing will be needed to resolve whether the interrupted coding sequence constitutes a bona fide mutation in the NJAY lineage.

Although a number of potential genetic differences between NJAY and AY-WB were observed, including those that may be due to lineage-specific gains (or losses) of proteins, these results should not yet be considered conclusive, in particular given the potential for errors in genome sequencing, assembly and/or annotation. Further investigation using molecular methods could be utilized to more rigorously test such preliminary findings based on the draft genome sequence presented here. Notwithstanding these ordinary technical limitations of genomics technology, however, highly similar matches between protein sequences offer compelling evidence that the genes in question are in fact shared between the taxa with a high degree of conservation.

Genome sequence data produced in this study should constitute a useful resource for scientists investigating the genetic determinants underlying plant pathogenicity of the NJAY strain of ‘*Candidatus* Phytoplasma asteris’, as well as other closely related phytoplasma strains. NJAY phytoplasma has been maintained in periwinkle and propagated through grafting in the authors’ USDA-ARS greenhouse for more than 20 years. Various symptom changes have been observed during this period. The varying symptoms exhibited by periwinkles infected with NJAY may be due to population-specific genotypic differences in various graft-isolates in different time periods. The sequence resources and comparative genomics findings reported here are far from conclusive to prove this intriguing hypothesis. Nevertheless, they should be of considerable use for initiating further studies.

## Materials and methods

NJAY phytoplasma strain in periwinkle was originally obtained from Dr. C. J. Chen, emeritus professor at Rutgers University in New Jersey, and maintained in the USDA periwinkle collection. This phytoplasma strain was insect transmitted from aster yellows phytoplasma infected lettuce to periwinkle [23]. NJAY phytoplasma DNA was extracted from sieve cell preparations isolated from infected periwinkle plants (*Catharanthus roseus*) exhibiting aster yellows syndrome, including typical phyllody and witches’-broom symptoms, as previously described [27,28] with the addition of RNase A digestion prior to the final phenol-chloroform extraction. These materials were used to prepare a DNA library for use with 454 sequencing instruments. Assemblies for ‘*Ca*. Phytoplasma asteris’ strains AY-WB (GenBank accession no, GCA_000012225.1) and OY-M (Genbank accession no. GCA_000009845.1), as well as ‘*Candidatus* Phytoplasma australiense’ strains PAa (GenBank accession no. GCA_000069925.1) and SLY (GenBank accession no. GCA_000397185.1), were obtained [5,6,8,9]. Using each of these as references, a chromosomal assembly of NJAY was prepared from unassembled 454 reads using Roche’s runMapping utility (version 2.9) with default parameter settings [29] and a 500 bp floor imposed on resultant contig length. Because the sequencing library consisted of a mixture of DNA from both phytoplasma and the host plant, a reference-based assembly strategy was utilized to minimize the chances of unintentional and erroneous incorporation of viridiplantae sequences into the NJAY genome.

Average nucleotide identity estimates were calculated at the whole-genome level using the ANIb method implemented in JSpecies [30]. Using ‘*Candidatus* Phytoplasma pruni’ strain CX (Genbank accession no. GCA_001277135.1; [17]) as an outgroup, a neighbor-joining tree was computed and rendered using the neighbor and drawgram programs of PHYLIP, respectively [31].

The Prodigal prokaryotic *ab initio* gene finding system [32] was used to identify protein coding genes in genomic sequence data, which were compared with known proteins in the NCBI NR database using blastp [33] with an E-value cutoff of 1E-15. Annotated proteins associated with the genomes for all other phytoplasma strains considered in this study were retrieved from GenBank and pooled with those identified in NJAY. Using a 65% similarity threshold, these sequences were clustered using the cd-hit program [34]. Independently for each cluster, protein sequences were retrieved and multiply aligned using T-Coffee [35]; clusters whose constitutent sequence lengths exhibited a sample standard deviation of ≥4.0 were discarded, unless scrutinous manual inspection suggested they be retained. Multiple sequence alignment results were manually refined and then used to guide alignment of underlying coding sequences with EMBOSS’ tranalign utility [36]. Aligned coding sequences were concatenated, and a Bayesian phylogeny was produced using the codon-based, haploid model implemented in MrBayes [37,38]. The consensus tree was visualized using FigTree v1.4.1 (http://tree.bio.ed.ac.uk/software/figtree/).

To identify putative plasmids harbored by NJAY, a set of four known plasmids from the AY-WB strain was obtained for referential use, these having accession numbers NC_007717 (i.e., “pAYWB-I”), NC_007718 (“pAYWB-II”), NC_007719 (“pAYWB-III”) and NC_007720 (“pAYWB-IV”) [6]. Reference-based assemblies were prepared for each of these plasmids using the Roche runMapping utility with default parameter settings, and resulting NJAY contigs were compared to their respective AY-WB plasmid reference using blastn. Similarly, residues 195,949 through 216,041 of the AY-WB chromosome (GenBank accession no. 84789385) were extracted and used as a reference for assembly; this segment corresponds to a known mobile element, dubbed “PMU1” (for potential mobile unit-1), in AY-WB [39].

Using the needle utility from EMBOSS with default parameter settings, global protein alignments were computed between all genes identified in NJAY and all secreted, membrane-bound proteins (SMPs), as well as against all secreted proteins released as soluble biomolecules (SAPs); these reference proteins correspond to the chromosome-encoded subset of genes listed in Tables 1 and 2, respectively, of Bai et al. [25]. Optimal-scoring matches were identified and categorized on the basis of percent similarity: “highly similar” implied ≥ 95.0%, “moderately similar” between 95.0 and 80.0%, and “somewhat similar” < 80.0%. Secreted NJAY proteins were scanned for potential secretion signals using Phobius [40] and the combination of SignalP with TMHMM (using gram-positive, HMM-based parameter settings) [41].

Shared metabolic capacity among NJAY, AY-WB and OY-M was determined in two manners: the first utilized existing annotations for AY-WB and OY-M, and the second involved reannotating genic content for these two reference taxa. Prodigal-predicted NJAY protein sequences were annotated using Blast2GO [42], from which Enzyme Commission (EC) numbers were extracted and compared in pairwise fashion with each of AY-WB and OY-M using the EC2KEGG program [43]. The Prodigal gene finder was then run independently on AY-WB and OY-M, and resultant protein sequences were assigned EC numbers using Blast2GO. The species-specific EC lists obtained were compared and contrasted manually.

## Supporting information

S1 Table Syntenic regions observed between the chromosomal genome of NJAY and that of the AY-WB and OY-M reference sequences used to guide assembly. (XLSX)

S2 Table Blast search results of six protein coding genes found in NJAY, but not present in the annotated protein sets for AY-WB, OY-M, PAa, SLY or CX available at GenBank per blastp. (XLSX)

S3 Table Enzyme Commission (EC) codes identified among NJAY, AY-WB and OY-M using annotations currently available in KEGG per EC2KEGG, as well as those obtained following reannotation of protein complements using Prodigal and Blast2GO. (XLSX)
